# Identification of essential amino acid residues in the nisin dehydratase NisB

**DOI:** 10.3389/fmicb.2015.00102

**Published:** 2015-02-26

**Authors:** Rustem Khusainov, Auke J. van Heel, Jacek Lubelski, Gert N. Moll, Oscar P. Kuipers

**Affiliations:** ^1^Department of Molecular Genetics, Groningen Biomolecular Sciences and Biotechnology Institute, University of GroningenGroningen, Netherlands; ^2^LanthioPharmaGroningen, Netherlands; ^3^Kluyver Centre for Genomics of Industrial FermentationGroningen, Netherlands

**Keywords:** NisB, dehydratase, posttranslational modifications, mechanism, NisC, lanthionine, lantibiotics, nisin

## Abstract

Nisin is a posttranslationally-modified antimicrobial peptide that has the ability to induce its own biosynthesis. Serines and threonines in the modifiable core peptide part of precursor nisin are dehydrated to dehydroalanines and dehydrobutyrines by the dehydratase NisB, and subsequently cysteines are coupled to the dehydroamino acids by the cyclase NisC. In this study, we applied extensive site-directed mutagenesis, together with direct binding studies, to investigate the molecular mechanism of the dehydratase NisB. We use a natural nisin-producing strain as a host to probe mutant-NisB functionality. Importantly, we are able to differentiate between intracellular and secreted fully dehydrated precursor nisin, enabling investigation of the NisB properties needed for the release of dehydrated precursor nisin to its devoted secretion system NisT. We report that single amino acid substitutions of conserved residues, i.e., R83A, R83M, and R87A result in incomplete dehydration of precursor nisin and prevention of secretion. Single point NisB mutants Y80F and H961A, result in a complete lack of dehydration of precursor nisin, but do not abrogate precursor nisin binding. The data indicate that residues Y80 and H961 are directly involved in catalysis, fitting well with their position in the recently published 3D-structure of NisB. We confirm, by *in vivo* studies, results that were previously obtained from *in vitro* experiments and NisB structure elucidation and show that previous findings translate well to effects seen in the original production host.

## Introduction

Lantibiotics are ribosomally synthesized polycyclic peptides. The rings contain post-translationally introduced thioether-bridged amino acids, so called lanthionines. The most studied lantibiotic, which has also found commercial application, is nisin. Nisin has been successfully used for over 50 years as a food preservative without significant resistance development in food pathogens (Gravesen et al., 2001; Kramer et al., 2008).

**Figure 1:**
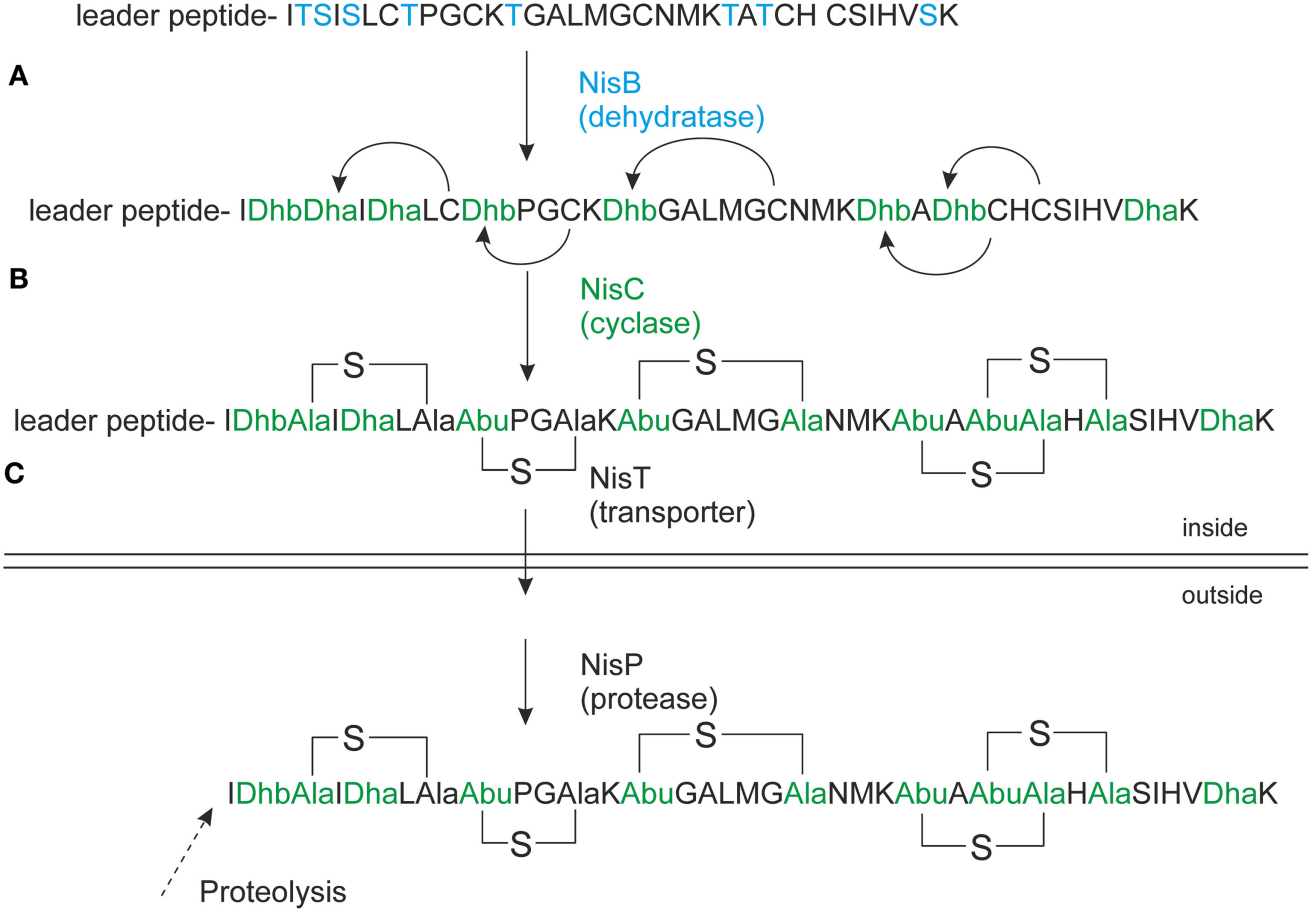
**Nisin biosynthesis**. NisA is ribosomally synthesized in a form of precursor nisin. **(A)** NisB dehydrates underlined Ser/Thr's (in bold), the resulted dehydrated precursor nisin contains dehydroalanines (Dha) and dehydrobutyrines (Dhb); **(B)** NisC forms thioether bridges between dehydrated residues and cysteines resulting in fully modified precursor nisin. **(C)** NisT transports the fully modified precursor nisin outside the cell. NisP cleaves off the nisin leader extracellularly to liberate active nisin. The Figure is in accordance with recent recommendations for a universal nomenclature for ribosomally synthetized and posttranslationally modified peptide natural products (Arnison et al., 2013).

Precursor nisin is composed of a 23 amino acid leader peptide followed by a modifiable 34 amino acid core peptide part (Figure 1). The leader peptide is a recognition signal for the modification enzymes NisB and NisC (Xie et al., 2004; Mavaro et al., 2011; Khusainov et al., 2013a) and the transporter NisT (van der Meer et al., 1994). It furthermore keeps the fully modified precursor nisin inactive (Kuipers et al., 1993b; van der Meer et al., 1994). Nisin contains one lanthionine ring and four (methyl)lanthionine rings that are introduced enzymatically. Analysis of truncated nisin variants has shown that the presence of at least the three N-terminal rings ABC is necessary for nisin variants to exert some antimicrobial activity (Chan et al., 1996).

NisB is a dehydratase of about 117.5 kDa (Kuipers et al., 1993a). It is the first enzyme to come into play during the modification by dehydrating serines and threonines in the core peptide part of precursor nisin, to form dehydroalanines and dehydrobutyrines, respectively (Figure 1). Moreover, *in vitro* activity studies have indicated a possible mechanism for the dehydration reaction, involving glutamylination of Ser and Thr residues (Garg et al., 2013). Recently the structure of NisB was solved implicating an un-expected cofactor namely glutamyl-tRNA^Glu^ (Ortega et al., 2015). This study gives insight in the mechanism of action of NisB and also revealed the location of its active sites, the glutamylation domain and the glutamate elimination domain. Dehydrated amino acids are coupled to cysteines by a second enzyme, NisC, in a regio- and stereospecific manner, to generate lanthionine rings (Figure 1). A model of the catalytic mechanism of NisC has been proposed based on *in vitro* studies and the crystal structure of NisC (Li and van der Donk, 2007).

Modified precursor nisin is transported via the dedicated ABC transporter NisT and the nisin leader peptide is extracellularly cleaved off by the protease NisP, liberating active nisin (Kuipers et al., 1993a). While nisin itself is renowned for its strong antimicrobial and autoinducer activity, the nisin modification enzymes have additional relevance because of their influence on the extent of modification. NisB, NisC, and NisT can also modify and transport peptides unrelated to nisin provided that the nisin leader peptide is present at the N-terminus (Kuipers et al., 2004; Kluskens et al., 2005; Rink et al., 2007; Majchrzykiewicz et al., 2010; van Heel et al., 2013). In this way lanthionines can be introduced into medically relevant peptides. By imposing a conformational constraint, the lanthionines confer resistance to breakdown by peptidases (Rink et al., 2010), enable in specific cases oral and pulmonary delivery (de Vries et al., 2010) and allow to select for peptides with optimal receptor interaction, thus strongly enhancing their therapeutic potential (Kluskens et al., 2005, 2009; van Heel et al., 2011). LanB enzymes do not share significant sequence homology to members of known protein families, thus representing a unique family of enzymes. Recent studies have shown that the process of dehydration by NisB and cyclization by NisC can alternate at the nisin precursor peptide (Kuipers et al., 2008; Lubelski et al., 2009). This appears to proceed from the N- to the C-terminus for class I enzymes (Lubelski et al., 2009), as well as for class II enzymes (Lee et al., 2009). A complex of the nisin biosynthesis enzymes has been isolated consisting of NisB, NisC, and NisT (Khusainov et al., 2011). Moreover, NisB has been demonstrated to have stronger interactions with precursor nisin than NisC has (Khusainov et al., 2011). Interestingly, the nisin leader is not absolutely required for class I lantibiotic biosynthesis *in vivo* (Khusainov and Kuipers, 2012), however, its addition *in trans* increases the efficiency of modification (Khusainov and Kuipers, 2012). Recently, it has been shown that synthetic nisin variants lacking Ser/Thr's in the core structure still bind NisB and synthetic nisin variant lacking Cys in the core structure bind NisC (Khusainov and Kuipers, 2013b). Increasing the number of negatively charged amino acids in the core peptide part of precursor nisin does not abolish binding of the nisin modification enzymes to these unnatural nisin variants (Khusainov and Kuipers, 2013b).

Nisin's N-terminal lanthionine ring binds to the cell wall precursor lipid II that is considered to act as a docking molecule (Breukink et al., 1999; Hasper et al., 2006). Nisin exerts at least two modes of antimicrobial action: it displaces lipid II from the septum thereby inhibiting cell wall synthesis and it forms hybrid pores composed of nisin and lipid II, which permeabilize the target cell membrane (Hasper et al., 2006; Lubelski et al., 2008).

Four classes of lanthipeptides have been distinguished (Xie et al., 2004; Goto et al., 2010; Mueller et al., 2010). Nisin belongs to class I, in which precursor peptides are dehydrated by LanB enzymes and cyclized by LanC enzymes. (Methyl)lanthionines in classes II, III, and IV are installed by the bi- or multifunctional enzymes LctM, RamC/LabKC, or LanL, respectively, that perform both dehydration and cyclization reactions (Xie et al., 2004; Goto et al., 2010; Mueller et al., 2010). We here applied extensive protein engineering of NisB to elucidate the potential mechanistic roles of highly and less conserved residues. We identified two likely catalytic site residues, i.e., Y80 and H961 and several regions for substrate binding and discuss these results in conjunction with the recently published NisB 3D-structure (Ortega et al., 2015).

## Materials and methods

### Bacterial strains and growth conditions

Table S1 (supplementary material) lists the strains and plasmids that were used in this study. *Lactococcus lactis* was used as a host for the overexpression plasmids pNZnisA-E3 or pNZnisA-H6 expressing precursor nisin or His-tagged precursor nisin, respectively. Mutated versions of NisB as well as of wild type NisC and NisT were overexpressed using the pIL3BTC plasmid (Rink et al., 2005). Cells were grown as described previously (Khusainov et al., 2011) at 30°C in M17 medium (Difco) supplemented with 0.5% (w/v) glucose and antibiotics at 5 μg/ml chloramphenicol and 5 μg/ml erythromycin, where appropriate. When both chloramphenicol and erythromycin were used, 4 μg/ml of each was applied. Prior to mass spectrometric analyses, cells were cultured in minimal medium as previously described (Rink et al., 2005).

### Recombinant DNA techniques

Standard genetic manipulations were essentially performed as described by Sambrook and Russell (2001). Plasmid pIL3BTC (Rink et al., 2005) served as a template for PCR in order to obtain site-specific NisB mutants. The round PCR method was performed as described earlier (Rink et al., 2005). In brief, the primers used were 5′-phosphorylated to allow ligation of the amplicon ends after PCR. The primers were oriented in the reverse direction to allow amplification of the whole plasmid. The mismatches were in the 5′-ends of either the forward or the reverse primer. Standard PCR was performed with these primers according to the Phusion DNA-polymerase manufacture (Finnzymes). After PCR, the PCR product was purified with the PCR-purification kit (Roche). Subsequently, DNA ligation was performed with T4 DNA ligase (Thermo Scientific). Plasmid isolation was performed by means of the Plasmid DNA Isolation Kit (Roche Applied Science). Restriction analysis was performed with restriction enzymes from Thermo Scientific.

### Protein expression and purification

C-terminal His-tagged precursor nisin was purified as described before (Khusainov et al., 2011). *L. lactis* NZ9000 (de Ruyter et al., 1996) containing mutated versions of *nisB* together with wild type *nisTC* and *nisA* containing the C-terminal sequence for the His-tag, was grown overnight followed by 1:50 dilution in GM17 (M17 (Difco) supplemented with 0.5% (w/v) Glucose). Growth was continued until OD_660_ = 0.6, followed by induction with 0.5 ng/ml of nisin for 2 h. Cells were collected by centrifugation, and lysed by use of 10 μg ml^−1^ freshly prepared lysozyme solution, followed by the addition of 10 mM MgSO_4_ and 100 μg ml^−1^ Dnase I (Sigma). Cells were disrupted by several rounds of freeze thaw cycles with liquid nitrogen in cases when 0.5 L of culture was used. Cells were disrupted by French Pressure treatment (15,400 psi) in case 2 L cultures were used, and remaining debris was removed by low speed centrifugation (13,000 × g for 15 min at 4°C; Sorvall SS34 rotor).

### Mass spectrometric analysis

In order to conduct mass spectrometric analysis of the produced peptides we used crude supernatants from bacteria grown on minimal medium. Prior to the mass spectrometric analysis, samples were ZipTipped (C18 ZipTip, Millipore) essentially as described before (Khusainov et al., 2011). In short, ZipTips were equilibrated with 100% acetonitrile and washed with 0.1% trifluoroacetic acid. Subsequently, the supernatant containing the peptides was mixed with 0.1% trifluoroacetic acid and applied to a ZipTip. Bound peptides were washed with 0.2% trifluoroacetic acid and eluted with 50% acetonitrile and 0.1% trifluoroacetic acid. The eluent was mixed in a ratio of 1:1 with matrix (10 mg/ml α-cyano-4-hydroxycinnamic acid) and 1.5 μl was spotted on the target and allowed to dry. Mass spectra were recorded with a Voyager-DE Pro (Applied Biosystems) MALDI-time-of-flight mass spectrometer. In order to increase the sensitivity and the accuracy, external calibration was applied with six different peptides (Protein MALDI-MS Calibration Kit, Sigma).

### Interaction analysis of precursor nisin with the modification enzymes NisB and NisC

To investigate the binding of NisB mutants to precursor nisin a previously described pull-down method of the nisin biosynthesis complex, using His-tagged precursor nisin as bait, was applied (Khusainov et al., 2011).

## Results

**Figure 2:**
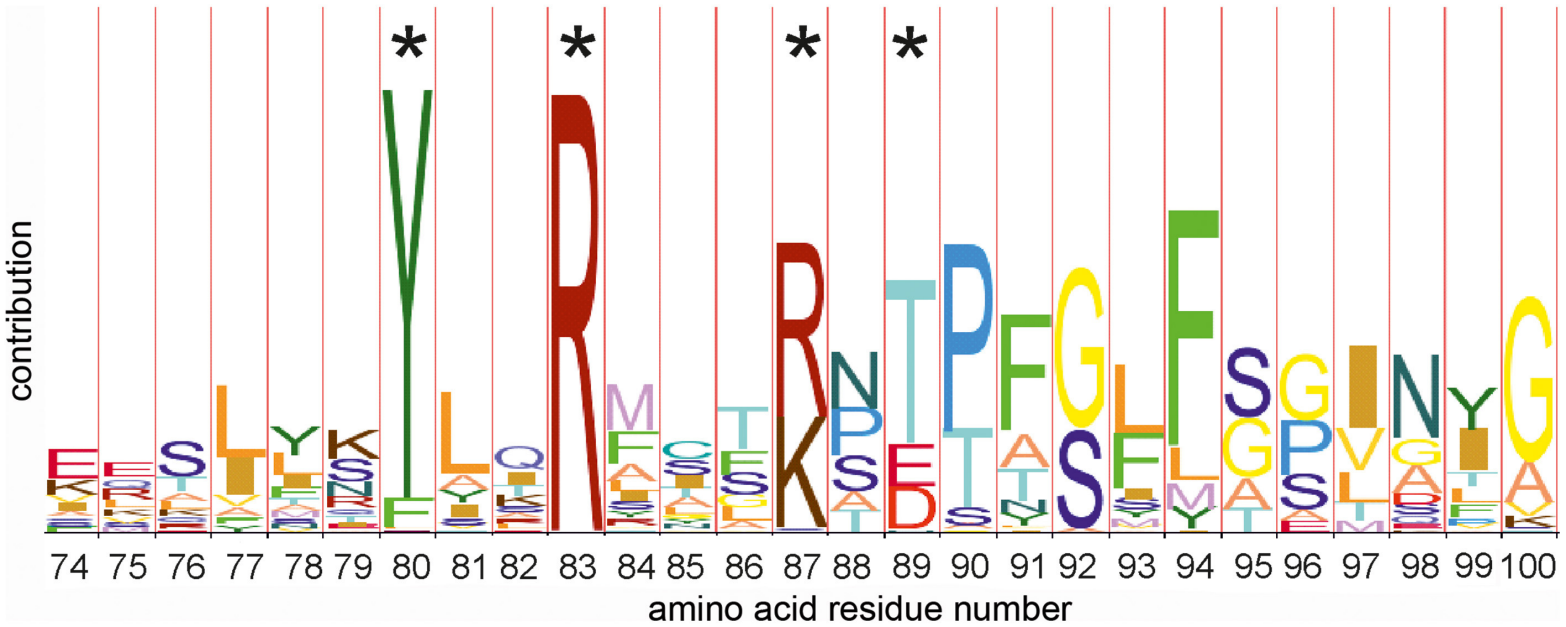
**HMM-logo of the N-terminal amino acid alignment of thirty six members of the dehydratase LanB family**. The hidden markov model (HMM) shows a number of conserved amino acid residues. Residues marked with an asterisk have been (among others) mutated in this study. Other mutations have been selected on the basis of the full models of the C (PFAM PF04738 http://pfam.xfam.org/family/PF04738) and the N terminal domains (PFAM PF04737 http://pfam.xfam.org/family/Lant_dehyd_N).

Amino acid sequence alignment of 36 LanB protein sequences resulted in the identification of several conserved residues (Figure 2) (Schuster-Bockler et al., 2004). We selected 25 (semi) conserved residues for site-directed mutagenesis. NisB mutants harboring single amino acid substitutions residues were generated (Table 1). The expression of the NisB mutants and their integrity were checked by, either mass spectrometric determination of nisin in the supernatant, or by SDS-PAGE and Western blot analysis (Figure S1). NisB is about 117.5 kDa and is known to have a natural N-terminal degradation product of ~90 kDa (Khusainov et al., 2011).

**Table 1:** **Dehydration pattern of precursor nisin modified by NisB mutants**.

**Mutated residue in NisB**	Dehydrations observed^*^	**Secretion of NisA**
(L223A, I224A), I298A, F342A, Y346F, P639A, R775A, Y827F, D843A, S844A, S958A, R966A, E975Q,	**8**, 7, (6)	^+^
D121A, D299A, D648A	8, **7**, (6)	^+^
T89A	7, **6**, 5	^+^
R784A	**7**	^+^
R83A, R83M	0, 1, 2, (3)	−
R87A	4, 5, 6, 7	−
R14A, W616A (Khusainov et al., 2011)	0, 1, 2, 3, 4, 5, 6, 7	−
Y80F, H961A	0	−

+ *Demonstrated by the NisA-H6 pull-down assay (Figure S1)*.

* *Most prominent mass peak observed is indicated with the bold number*.

**Figure 3:**
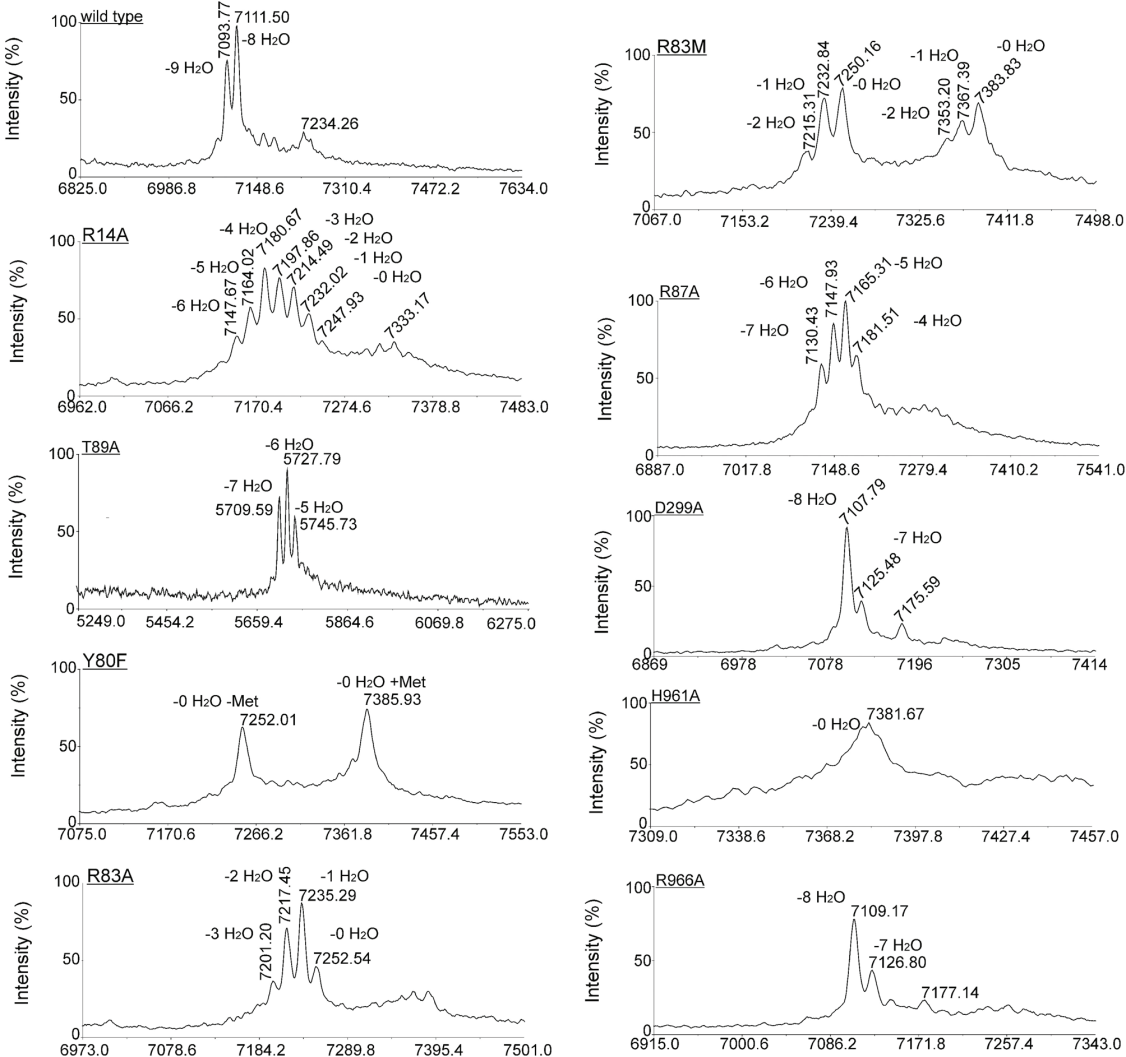
**Effect of NisB mutagenesis on the extent and pattern of dehydration of precursor nisin**. Matrix-assisted laser desorption ionization–time-of-flight (MALDI-TOF MS) spectra were obtained of Ni-NTA purified precursor nisin mutants containing the C-terminal extension GSIEGR followed by His6 tag, and modified by NisB mutants *in vivo*. In the case of mutants of NisB that resulted in a lack of secretion (Table 1), the His-tagged version of precursor nisin was purified out of the cytoplasm of the cell. Cells containing plasmid pIL3BTC and a plasmid encoding for NisA with C-terminal extension and a His6 tag were grown until OD 0.6, induced with 0.5 ng/ml nisin and let grow for two additional hours. Subsequently, cells were harvested, disrupted by French press and purified precursor nisin was analyzed by MALDI-TOF MS.

*L. lactis* strain NZ9000, expressing simultaneously *nisA, nisTC* and in each case a different mutant version of the *nisB* gene, was used to study the functionality of mutants of NisB. Wild type precursor nisin is naturally secreted out of the cell by NisT. In this study, the secreted precursor nisin variants were purified from the supernatant by ZipTip purification (Millipore) and subsequently analyzed by MALDI-TOF mass spectrometry (Tables 1, S2, Figure 3). Those precursor nisin variants that were not secreted, were His-tagged and Ni-NTA purified and subsequently analyzed by MALDI-TOF mass spectrometry (Tables 1, S2, Figure 3).

### Mutations in NisB that have no major impact on the secretion and modification of nisin

To gain insight in the mechanism of action of NisB 25 different mutations were made in the sequence. The mutations were selected to modify different conserved residues based on an alignment of LanB-type dehydratases. Throughout the sequence of NisB, we observed several repetitions of conserved leucine and isoleucine residues or islands of adjacent leucine-isoleucine residues. To investigate the role of these conserved leucine and isoleucine residues, we performed a substitution of a single isoleucine (I298A) and a double substitution of adjacent leucine-isoleucine residues (L223A, I224A). Both mutants resulted in the secretion of fully modified nisin. Further NisB mutants F342A, Y346F, D648A, P639A, R775A, Y827F, D843A, S844A, S958A, R966A, E975Q also resulted in the secretion of fully modified precursor nisin identical to the wild type precursor nisin. In all these samples the most prominent mass peak corresponded to the 8-fold dehydrated nisin as is normal for the wild type nisin. Therefore, these mutations appear not to affect NisB-precursor nisin interactions and NisB-catalyzed dehydration. The dehydration pattern of precursor nisin, modified and secreted by wild type NisBTC enzymes, harbors 8, 7, and 6 dehydrated residues, with 8 being the most predominant peak in the mass spectra. For the mutant R784A, the only peak observed corresponded to the 7 times dehydrated NisA, indicating a slightly reduced modification efficiency and possibly a slightly lower production level since no peaks corresponding to the 6 and 8 times dehydrated substrate were observed. The analyzed dehydration pattern of precursor nisin which was modified by the NisB mutants where aspartate was changed to alanine (D121A, D299A, D648A) showed a similar pattern to that of wild type. Although all three expected dehydration species were present, i.e., 8, 7 and 6, the major peak observed corresponded, unlike in wild type precursor nisin, to a 7-fold dehydrated precursor nisin. This indicates that the dehydration efficiency of the above Asp?Ala NisB mutants was slightly decreased.

Intriguingly, the mutation T89A led to a secreted precursor nisin containing exactly one dehydration less than usually observed for wild type NisB. Mass spectrometry demonstrated a dehydration pattern of precursor nisin with 7, 6 and 5 dehydrations, while the 6-fold dehydration peak was the most prominent peak (Table 1, Figure 3). T89 is not very remote from the likely catalytic residue Y80 (vide infra).

### Mutations in NisB that abolish secretion of modified precursor nisin (Table 1)

Notably, several single NisB mutations at conserved positions, i.e., R14A, Y80F, R83A, R83M, R87A, H961A (Table 1; Figure S1) and the previously reported W616A (Khusainov et al., 2011) resulted in a lack of secretion of modified precursor nisin to the outside of the cell, thus hampering the evaluation of mutant NisB-mediated dehydration. To analyze these intracellularly-trapped precursor nisins, His-tagged precursor nisin variants were constructed, used and purified by Ni-NTA columns from the cell extract (Figure S1). To break the cells (Figure S1), we used several rounds of liquid nitrogen freeze-thaw cycles, which lead to cell lysis. However, application of this method may result in differences in the efficiency of *L. lactis* cell lysis. For this reason, results presented here should be interpreted qualitatively only. Subsequently, these modified precursor nisin mutants were analyzed by MALDI-TOF mass spectrometry (Tables 1, S1, Figure 3). A previously developed pull-out assay, which relies on interaction of His-tagged precursor nisin with its modification enzymes (Khusainov et al., 2011) demonstrated that all these NisB mutants could still bind precursor nisin (Figure S1). Since unmodified precursor nisin can also be exported via NisT (Kuipers et al., 2004), the NisB mutants leading to intracellularly trapped precursor nisin apparently have a reduced capacity to release their substrate. Furthermore, these data clearly demonstrate that the specific NisB mutants that cause a lack of secretion of precursor nisin, have a reduced dehydration capacity.

### NisB single mutants R83A, R83M, and R87A have severely reduced dehydration capacities

Interestingly, R83A and R83M were severely hampered in their dehydration capacity and led to intermediate dehydration patterns: up to 3 or 2-fold dehydration of precursor nisin. R87A resulted in 4, 5, 6, and 7 dehydrations. This suggests that these residues are important for possible positioning of the partially dehydrated peptide into the active site.

### Catalytic residues of NisB

Site-directed mutagenesis of Y80F and H961A resulted in a lack of secreted precursor nisin. This result is consistent with recent studies (Garg et al., 2013; Ortega et al., 2015), where mutagenesis of the NisB H961 residue also resulted in a non-dehydrated precursor nisin and was shown to be part of the active site of the glutamate elimination domain. Applying the previously described modification enzyme co-purification binding assay (Khusainov et al., 2011) we showed that the NisB mutants Y80F and H961A are still able to bind precursor nisin (Figure S1). MALDI-TOF MS analysis resulted in one major peak, corresponding to fully unmodified precursor nisin for both NisB mutants (Table S2, Figure 3). These results strongly suggest that Y80 and H961 are directly involved in catalysis, since no dehydration at all was observed.

## Discussion

The class I dehydratase NisB is a remarkable catalyst that breaks 16 bonds by modifying 3 Ser and 5 Thr residues in precursor nisin. To investigate the effect of mutations in NisB on its activity, we applied extensive site-directed mutagenesis of its conserved residues without prior knowledge of its later published structure. This resulted in the identification of residues in NisB that are important for catalysis and/or for the efficiency of dehydration.

The effects of the mutations that we observed can be classified into three groups: (1) mutations that resulted in a wild type extent of dehydration and secretion, (2) mutations that resulted in non-secreted peptides with intermediate dehydration patterns, and (3) mutations that resulted in non-secreted and unmodified precursor nisin.

The NisB mutants from the groups 2 and 3 prevented export of precursor nisin. However, NisT has been demonstrated of being capable of exporting unmodified precursor nisin in the absence of NisB (Kuipers et al., 2004). It can be speculated that the absence of the export might be caused by strongly reduced release of precursor nisin from a mutant NisB. Another explanation might be that lack of secretion is observed because NisB and NisC are acting alternatingly (Lubelski et al., 2009). Incomplete dehydration might disturb this delicate process leading to complexes that do not release the product, which might block the export.

Mechanistic *in vitro* investigations of the dehydration reaction of the bifunctional and multifunctional LctM, RamC/LabKC, and LanL enzymes demonstrated that LctM, RamC/LabKC, and LanL phosphorylate Ser and Thr in the substrate peptide, as was evidenced by MALDI-TOF MS, which identified peaks with a mass shift of +80 Da differences (Chatterjee et al., 2005; Goto et al., 2010; Mueller et al., 2010). The class II LanM enzymes have been shown to use ATP as an energy source. Notably, the class III labyrinthopeptin A2 modification enzyme LabKC has been recently demonstrated to require GTP for the phosphorylation and dehydration reaction of serines (Mueller et al., 2010). The recently published reconstitution of the *in vitro* activity of class I NisB (Garg et al., 2013), shows that the dehydration by class I lantibiotic enzymes happens via glutamylation of Ser/Thr and not by phosphorylation. It is not clear why class I lantibiotic enzymes use different mechanism for dehydration, however this might be due different evolutionary lineages that these enzymes followed.

In the *in vitro* study of Garg et al., individual replacement of residues Arg14, Arg83, Arg87, Thr89, Asp121, Asp299, Arg464, and Arg966 with Ala and subsequent expression and purification of these NisB mutants in *E. coli* resulted in abolishment of dehydration (Garg et al., 2013). In our *in vivo* study in its native host *L. lactis*, the NisB mutants Arg14, Arg83, Arg87, Thr89, Asp121, Asp299, and Arg966 resulted in partial dehydration of the precursor nisin (Table 1). These differences are most likely due to the differences in the host (*E. coli* vs. *L. lactis*) or due to the differences between *in vivo* and *in vitro* conditions. However, both of the studies pinpoint the importance of these residues for the dehydration reaction. Moreover, we identified one more residue of crucial importance: i.e., Y80. The mutant Y80F most likely interferes with the glutamylation domain that was recently identified (Ortega et al., 2015). Furthermore, we show that NisB R14A, Y80F, R83A, R83M, R87A, H961A, and W616A mutants result in a lack of transport of precursor nisin.

**Figure 4:**
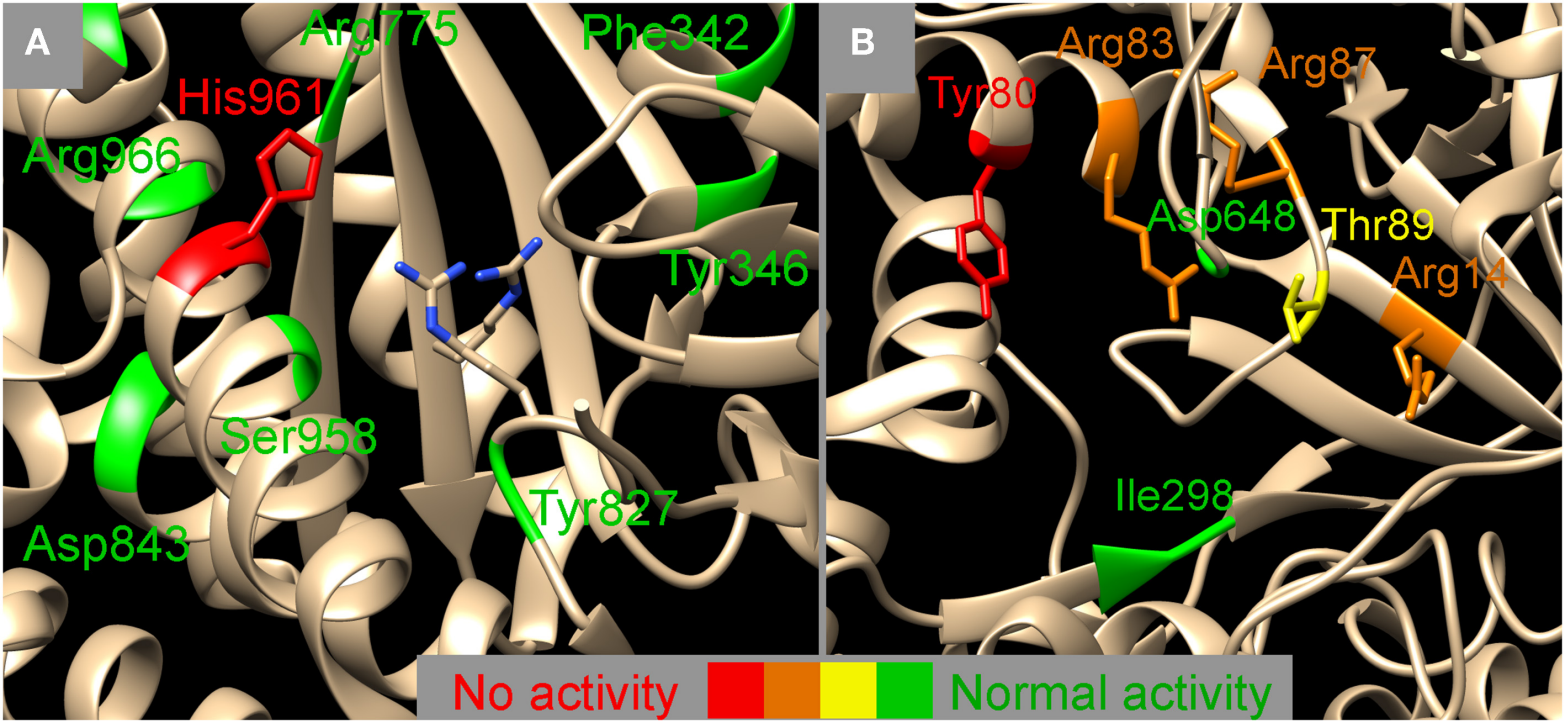
**Mapping the NisB mutants on two functional domains, the glutamylation domain (**A)** and the glutamate elimination domain (**B**) (PDB 4WD9, Ortega et al., 2015)**. Colors indicate the effect of the mutation on the dehydratase activity, red, no activity observed; orange, activity severely hampered; yellow, activity slightly hampered; green, normal activity. The exact nature of the mutation can be looked up in Table 1. **(A)** Next to the sidechain of His961 also the sidechains of Arg826 and Arg786 are indicated which have been indicated to be important for glutamylation previously (Garg et al., 2013). The vicinity of the glutamylation domain (**A**) seems to allow for some amino acid changes whereas the surrounding of the glutamate elimination domain (**B**) seems to be more strictly determined. Image was created using UCSF chimera version 1.10.1 (Pettersen et al., 2004).

Here we present data that is perfectly in line with the recent publication of the structure of NisB (Ortega et al., 2015) as can be seen in Figure 4, indicating the relation between position and effect of the mutation in the 3D-structure of NisB. With our *in-vivo* results we can confirm conclusions made on the basis of experiments that were performed *in vitro* using heterologously expressed enzymes and substrates.

In the structure of NisB four specific domains have been identified, a glutamylation domain, a glutamate elimination domain, a tRNA interaction domain and a region likely to interact with the nisin and its leader. We investigated several mutants that lie within the proximity of the glutamylation domain (Figure 4A), of which only H961A resulted in complete loss of activity. All other mutants showed normal dehydration patterns indicating a certain degree of structural freedom around the active site. The glutamate elimination domain shows a different picture (Figure 4B). The mutant Y80F resulted in total loss of activity but also many mutations in the vicinity (R83A/M, R87A, R14A, and T89A) had a detrimental effect on the activity. Although in Figure 4B, D648 seems to be in the proximity of the active site, this is actually not the case (2D vs. 3D artifact) and therefore it is explainable that mutating it into Ala had no effect on the activity. The glutamate elimination site contains several conserved residues that are less tolerant to amino acid changes. No mutants close to the tRNA interaction domain were investigated. Close to the putative nisin leader interaction site (<10Ǻ) only one double mutant (I223A, I224A) was investigated which resulted in normal activity. Although the NisA interaction region of the structure was not extensively probed in this study, it can be expected that there is a high degree of tolerance to amino acid substitutions since many different substrates can be modified by NisB.

From the mutants we tested only the single mutants, i.e., NisB Y80F and NisB H961A result in non-modified precursor nisin, and importantly these mutants still bind precursor nisin and are able to co-purify NisB in the precursor nisin co-purification assay. Overall, we can conclude that R14, R83, R87, and W616 (Khusainov et al., 2011) in NisB play an important, though not direct catalytic role, in the dehydration reaction of class I lantibiotics, since their substitution leads to a reduced extent of dehydration, without completely abolishing it and a lack of secretion. The (novel) single point mutation Y80F and the single point mutation H961A in NisB lead to unmodified and non-secreted precursor nisin. Notably, NisB residues that resulted in an intermediate dehydration pattern also resulted in a lack of secretion of precursor nisin. This observation suggests that the NisB mutants that result in unsecreted precursor nisin either do not release the substrate precursor nisin or a subsequent cyclization reaction by NisC is significantly slowed down, preventing the export. This observation indicates that the modification and the transport processes are linked to each other, in line with a previous publication (van den Berg van Saparoea et al., 2008), possibly through the complex formation that has recently been described (Khusainov et al., 2011).

### Conflict of interest statement

The authors declare that the research was conducted in the absence of any commercial or financial relationships that could be construed as a potential conflict of interest.
