# Supplementary material for: Identification of essential amino acid residues in the nisin dehydratase NisB
Source: Front Microbiol. 2015 Feb 26;6:102. doi: 10.3389/fmicb.2015.00102 (PMC4341554; doi:10.3389/fmicb.2015.00102)
Supplement: Supplementary file 1 [file DataSheet1.DOCX]

**Supplemental figures and tables.**

**Table S1. *Lactococcus lactis* strain and plasmids used in the study.**

| Strain | Characteristics | References |
| --- | --- | --- |
| NZ9000 | *nisRK* | (de Ruyter *et al.*, 1996a) |
| Plasmids |  |  |
| pIL3BTC | *nisBTC*, encodes for nisin modification machinery | (Rink *et al.*, 2005) |
| pNZnisA-E3 | *nisA,* encoding for nisin | (Kuipers *et al.*, 2004) |
| pNZnisA-H6 | *nisA,* encoding for nisin with C-terminal his tag and Xa cleavage site | (Khusainov *et al.*, 2011) |
| pNGnisPT | *nisP, nisT*  encoding NisP and NisT | (Khusainov *et al.*, 2011) |
| pIL3BTC, NisB W616A | *nisB* encoding for NisB with W616A substitution | (Khusainov *et al.*, 2011) |
| pIL3BTC, NisB R14A | *nisB* encoding for NisB with R14A substitution | this study |
| pIL3BTC, NisB T89A | *nisB* encoding for NisB with T89A substitution | this study |
| pIL3BTC, NisB Y80F | *nisB* encoding for NisB with Y80F substitution | this study |
| pIL3BTC, NisB R83A | *nisB* encoding for NisB with R83A substitution | this study |
| pIL3BTC, NisB R83M | *nisB* encoding for NisB with R83M substitution | this study |
| pIL3BTC, NisB R87A | *nisB* encoding for NisB with R87A substitution | this study |
| pIL3BTC, NisB D121A | *nisB* encoding for NisB with D121A substitution | this study |
| pIL3BTC, NisB I298A | *nisB* encoding for NisB with I298A substitution | this study |
| pIL3BTC, NisB D299A | *nisB* encoding for NisB with D299A substitution | this study |
| pIL3BTC, NisB L223A,I224A | *nisB* encoding for NisB with double L223A,I224A substitution | this study |
| pIL3BTC, NisB F342A | *nisB* encoding for NisB with F342A substitution | this study |
| pIL3BTC, NisB Y346F | *nisB* encoding for NisB with Y346F substitution | this study |
| pIL3BTC, NisB P639A | *nisB* encoding for NisB with P639A substitution | this study |
| pIL3BTC, NisB E689A | *nisB* encoding for NisB with E689A substitution | this study |
| pIL3BTC, NisB R775A | *nisB* encoding for NisB with R775A substitution | this study |
| pIL3BTC, NisB R784A | *nisB* encoding for NisB with R784A ubstitution | this study |
| pIL3BTC, NisB R826A | *nisB* encoding for NisB with R826A substitution | this study |
| pIL3BTC, NisB Y827F | *nisB* encoding for NisB with Y827F substitution | this study |
| pIL3BTC, NisB D843A | *nisB* encoding for NisB with D843A substitution | this study |
| pIL3BTC, NisB S844A | *nisB* encoding for NisB with S844A S844A substitution | this study |
| pIL3BTC, NisB S958A | *nisB* encoding for NisB with S958A substitution | this study |
| pIL3BTC, NisB H961A | *nisB* encoding for NisB with H961A substitution | this study |
| pIL3BTC, NisB R966A | *nisB* encoding for NisB with R966A substitution | this study |
| pIL3BTC, NisB E975Q | *nisB* encoding for NisB with E975Q substitution | this study |

**Table S2. Dehydration of His-tag-isolated non-secreted precursor nisin by NisB mutants**

| NisB / NisB mutant | Dehydration extent | Observed (Da) | calculated * |
| --- | --- | --- | --- |
| Wild type NisB | 9  8 | 7093.77  7111.50 | 7092  7110 |
| R14A | 7  6  5  4  3  2  0 | 7131.79  7149.26  7165.61  7182.27  7199.46  7216.10  7249.55 | 7128  7146  7164  7182  7200  7218  7254 |
| T89A^‡^ | 7  6  5 | 5709.59  5727.79  5745.73 | 5706  5724  5742 |
| **Y80F** | **0** | 7252.01 | 7254 |
| R83A | 3  2  1  0 | 7201.20  7217.45  7235.29  7252.54 | 7200  7218  7236  7254 |
| R83M | 2  1  0 | 7215.31  7232.84  7250.16 | 7218  7236  7254 |
| R87A | 7  6  5  4 | 7129.04  7146.52  7163.88  7180.07 | 7128  7146  7164  7182 |
| D299A | 8  7 | 7107.79  7125.48 | 7110  7128 |
| **H961A** | **0** | 7381.67 | 7385 |
| R966A | 8  7  6  4 | 7109.17  7126.80  7145.02  7177.14 | 7110  7128  7146  7182 |

‡ wild-type precursor nisin without a His-tag was used

Figure S1A. SDS page of NisB pull downs


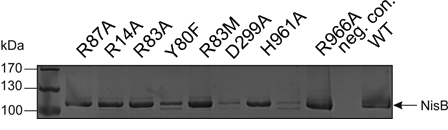


Figure S1B.


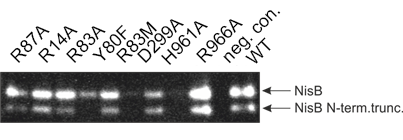


**Figure S1.** Pull-down assays were performed to analyze NisB mutants that resulted in no secretion of nisin. The secreting mutants D299A and R966A were added as a control. For the assay 0.5 L GM17 medium was inoculated with ON culture of *L. lactis* containing plasmid pIL3BTC and a plasmid encoding NisA with C-terminal extension, GSIEGR, and a His6-tag. Cultures were grown until OD 0.6, induced with 0.5 ng/ml nisin and incubated for an additional two hours. Subsequently, cells were harvested, disrupted by several cycles of freezing and defreezing in liquid nitrogen. Ni-NTA-purified precursor nisin from cell extracts was applied to SDS-PAGE, NisB co-purified with precursor nisin is visualized by anti-NisB antibodies on a Western blot. **(S1A)** SDS-PAGE analysis showing elutions from Ni-NTA purification of precursor nisin with the his-tag at the C-terminus of NisA precursor. **(S1B)** Western blot with Anti-NisB antibodies showing elutions from Ni-NTA purification of precursor nisin with the his-tag at the C-terminus of NisA precursor.
